# Retraction: JQ1 suppresses tumor growth through downregulating LDHA in ovarian cancer*

**DOI:** 10.18632/oncotarget.28864

**Published:** 2026-04-24

**Authors:** Haifeng Qiu, Amanda L. Jackson, Joshua E. Kilgore, Yan Zhong, Leo Li-Ying Chan, Paola A. Gehrig, Chunxiao Zhou, Victoria L. Bae-Jump

**Affiliations:** ^1^Department of Obsterics and Gynecology, The First Affiliated Hospital of Zhengzhou University, Zhengzhou, Henan 450052, China; ^2^Division of Gynecological Oncology, Department of Obstetrics and Gynecology, School of Medicine, University of North Carolina at Chapel Hill, Chapel Hill, NC 27599, USA; ^3^Division of Gynecological Oncology, Linyi Tumor Hospital, Linyi, Shandong 276001, China; ^4^Department of Technology R&D, Nexcelom Bioscience LLC, Lawrence, MA 01843, USA; ^5^Linberger Comprehensive Cancer Center, School of Medicine, University of North Carolina at Chapel Hill, Chapel Hill, NC 27599, USA; ^*^This work was partially presented at the 2013 Annual Meeting of the American Association for Cancer Research (AACR), Washington DC

**This article has been retracted:** An investigation by Oncotarget, prompted by comments on PubPeer, has identified internal duplications and image manipulation in the following figures:

- Figures 1B and 3D: The α-tubulin loading controls for Hey and SKOV3 cells in Figure 1B were reused in Figure 3D as controls for SKOV3 and Hey cells, respectively.
- Figure 5C: The four α-tubulin bands in the Hey cells western blot were found to be identical to those in the SKOV3 cells blot following image manipulation.

At the same time The University of North Carolina at Chapel Hill conducted a research misconduct proceeding that involved this article and concluded that research misconduct occurred, specifically the falsification and reuse of western blot bands from different experimental conditions in Figures 1B, 3D and 5C. Therefore, the results and conclusions of this publication are no longer reliable. The authors requested the article retraction. Taking into consideration all these facts the Editorial decision has been made to retract this article. All authors agree with this decision, with the exception of Dr. Haifeng Qiu, who did not respond, and Dr. Leo Li-Ying Chan, who could not be reached.

Original article: Oncotarget. 2015; 6:6915–6930. 6915-6930. https://doi.org/10.18632/oncotarget.3126

